# A Novel Nomogram to Predict Survival in Patients With Recurrence of Pancreatic Ductal Adenocarcinoma After Radical Resection

**DOI:** 10.3389/fonc.2020.01564

**Published:** 2020-09-03

**Authors:** Chaobin He, Shuxin Sun, Yu Zhang, Xiaojun Lin, Shengping Li

**Affiliations:** ^1^State Key Laboratory of Oncology in South China, Department of Pancreatobiliary Surgery, Collaborative Innovation Center for Cancer Medicine, Sun Yat-sen University Cancer Center, Guangzhou, China; ^2^State Key Laboratory of Ophthalmology, Zhongshan Ophthalmic Center, Sun Yat-sen University, Guangzhou, China

**Keywords:** pancreatic ductal adenocarcinoma, recurrence, surgery, nomogram, prognosis

## Abstract

The post-progression survival (PPS) of patients with pancreatic ductal adenocarcinoma (PDAC) after radical resection is varied and influenced by the characteristics of tumor progression. We aimed to establish and validate a nomogram to predict PPS for PDAC patients after surgery. A total of 302 PDAC patients who had undergone curative resection from 2008 to 2018 were enrolled in this study and randomly divided into training and validation cohorts at a ratio of 3:1. The nomogram was established based on independent prognostic factors selected by LASSO and Cox regression and measured by the area under the receiver operating characteristic curve (AUC) and the concordance index (C-index). Significant prognostic factors included carbohydrate antigen 19-9 (CA19-9), lymph node (LN)9 metastasis, LN14 metastasis, LN16 metastasis, tumor differentiation, imaging-detected tumor size, local progression, liver-only metastasis, lung-only metastasis, and multiple metastases. The nomogram built on these factors showed powerful efficacy in PPS prediction, with C-index values of 0.751 (95% CI 0.692–0.0.810) and 0.710 (95% CI 0.645–0.755) for the training and validation cohorts, respectively. The AUC values for the 1-year and 2-year PSS rates were 0.745, 0.747, and 0.783, 0.748, respectively; these values were higher than those of the 8th tumor–node–metastasis (TNM) stage system. The exploration of risk factors and the establishment of a nomogram can provide new versions of personalized recurrence management for PDAC patients after surgery.

## Introduction

Pancreatic ductal adenocarcinoma (PDAC) is a lethal disease with a 5-year overall survival (OS) rate of only 7% (1). Despite its low incidence, cancer-related deaths of PDAC patients rank fourth in the United States and continue to increase; thus, PDAC is expected to become the second-most common cause of cancer-related death by 2030 (2). Surgical resection, the only way of obtaining curative treatment of PDAC, is suitable for less than 20% of patients and improves the 5-year OS rate to 20–30% (3). Moreover, up to 80% of PDAC patients suffer recurrence soon after curative resection (4). Therefore, early recurrence poses a major challenge for the long-term survival of PDAC patients after curative resection.

Several stage systems have been used to estimate the OS or progression-free survival of PDAC patients (5, 6). These instruments were constructed on the basis of variables limited to primary tumor features. However, PDAC patients with varied progression patterns may have different rates of post-progression survival (PPS), which is greatly impacted by features of progression rather than primary tumor features (4, 7). Therefore, previously developed predictive systems may be less effective for PPS estimation in PDAC patients after surgery. Considering the absence of a predictive model specifically designed for PPS estimation, it was necessary to build a clinical prognostic predictive system to estimate PPS as well as recurrence after surgery in individual PDAC patients.

In the present study, we established a prognostic nomogram to predict the PPS of PDAC patients after curative resection. We also conducted comparisons of the efficacy of predicting survival prediction between this nomogram and a tumor–node–metastasis (TNM) system.

## Materials and Methods

### Patients

Consecutive PDAC patients who had undergone radical resection from 2008 to 2018 at Sun Yat-sen University Cancer Center (SYSUCC) were included in this study. The exclusion criteria were as follows: (1) distant metastasis before surgery, (2) history of a second tumor, (3) follow-up period <1 year, (4) missing information from follow-up records, and (5) microscopic or macroscopic incomplete resection. The margin for radical resection was defined as 1.5–2 mm, as in previous studies (8, 9). This study was approved by the Institutional Review Board of Sun Yat-sen University Cancer Center. All procedures involving human participants in the present study were performed in accordance with the ethical standards of institutional and/or national research committees as well as the 1964 Helsinki Declaration and its later amendments or similar ethical standards. Written informed consent was obtained from the patients prior to treatment.

### Data Collection

Resectability was judged by a pancreatic multidisciplinary team based on radiological examination, including computed tomography (CT), magnetic resonance imaging (MRI), and positron emission tomography/CT (PET-CT). Specialized pancreatic surgeons performed all radical resections of PDAC. An experienced pancreatic pathologist carried out the pathological diagnosis and description of the specimens, including such characteristics as tumor size, tumor differentiation, lymph node (LN) metastasis, LN total number, LN positive number, satellite foci, macrovascular and microvascular invasion, lymph vessels, and perineural and adjacent organ invasion. LN ratio (LNR) was defined as the proportion of positive LN in the total examined LN. Additionally, the associated radiological and clinical variables described in our previous studies (7) were included in the present study. All blood test indexes were obtained at the time at which tumor progression was diagnosed. Previously described (10) inflammation-based indexes, including the neutrophil-to-lymphocyte ratio (NLR), the platelet-to-lymphocyte ratio (PLR), the modified Glasgow Prognostic Score (mGPS), the prognostic nutritional index (PNI), the prognostic index (PI), and the systemic immune-inflammation index (SII), were analyzed as well.

### Recurrence Patterns

Information regarding the timing and pattern of recurrence was obtained at regular follow-up, which consisted of regular chest and abdominal CT, carbohydrate antigen 19-9 (CA19-9) measurement, and carcinoembryonic antigen (CEA) measurement every 3 months after surgery. Additional imaging modalities, such as MRI and PET/CT, were selectively performed to determine patterns of recurrence. When imaging findings were consistent with recurrence, biopsy was rarely performed. Otherwise, biopsy was conducted to confirm tumor progression or metastases. Either radiological or histological evidence was required for the diagnosis of disease recurrence. The date of the last follow-up occurred at the end of May 2019. The first location of recurrence was used to describe the recurrence patterns, which were categorized as in the study by Groot et al. (4). The cutoff value differentiating early and late progression was defined as 1 year following surgery (11). The terms liver-only and lung-only metastases referred to isolated hepatic and lung recurrence, respectively. The term others referred to isolated recurrence in other less common areas. Local recurrence and isolated distant metastasis occurring simultaneously were classified as local + distant while the term multiple referred to multiple distant metastases.

### Survival Outcomes and Statistical Analysis

Tumor progressions occurring within and beyond 1 year following surgery were classified as early and late progressions, respectively. Comparisons between the early and late progression groups were conducted for various clinical and pathological variables using chi-square analysis. The main survival outcome of this study was PPS, which was defined as the duration from the date of tumor progression to the date of death or the last date of follow-up. The Kaplan–Meier method was used to estimate survival. When the survival curves were not crossed, the survival differences were compared using a log-rank test. When the survival curves were crossed, the survival differences were further analyzed by landmark analysis. Multivariate analysis was adopted to determine significant prognosis factors based on the results of univariate analysis and the least absolute shrinkage and selection operator (LASSO) logistic regression model, which was used to explore the relationships between pathological and radiological variables and PPS. The area under the receiver operating characteristic (ROC) curves (AUCs) and concordance indexes (C-indexes) of the multimarker algorithms were calculated and compared with those of the TNM stage system. A two-tailed *P* < 0.05 was considered statistically significant. All statistical analyses were conducted using SPSS software version 22 (SPSS Inc., Chicago, IL, USA) and R software version 3.6.1 (R Development Core Team; http://www.r-project.org).

## Results

### Patients

A total of 355 PDAC patients had received radical resection from 2008 to 2018 at SYSUCC. Fifty-three patients were excluded from this study according to the exclusion criteria, including microscopic or macroscopic incomplete resection (10 patients), history of a second tumor (12 patients), and missing information from follow-up records (31 patients). Ultimately, 302 patients were included in the present study. Each patient was followed up for more than 1 year and the median follow-up time was 24.7 months [95% confidence interval (CI) 20.3–29.1 months]. During the follow-up period, a total of 173 (57.3%) patients developed tumor progressions after surgery. Comparisons between the early and late progression groups for clinical, pathological, and radiological variables are shown in Table 1. All patients were randomly divided into training (*n* = 227) and validation (*n* = 75) cohorts in a 3:1 ratio for the establishment and validation of the nomogram.

**Table 1:** Clinicopathological characteristics of patients with PDAC.

**Characteristics**		**Time to progression**					**Characteristics**		**Time to progression**				
		**Absence**	**Early progression**	**Late progression**	***N***	***P***			**Absence**	**Early progression**	**Late progression**	***N***	***P***
Whole cohort		129	129	44	302		Macrovascular invasion	Absence	120	114	39	273	0.408
Age	≤ 60 years	74	70	20	164	0.391		Presence	9	15	5	29	
	>60 years	55	59	24	138		Microvascular invasion	Absence	87	85	34	206	0.364
Gender	Male	53	46	20	119	0.453		Presence	42	44	10	96	
	Female	76	83	24	183		Lymph vessel invasion	Absence	65	55	20	140	0.199
Recurrence	Absence	129	24	21	174	<0.001		Presence	62	76	74	162	
	Presence	0	105	23	128		Perineural invasion	Absence	70	55	21	146	0.174
TNM stage	IA	33	10	11	54	0.001		Presence	59	74	23	156	
	IB	36	25	13	74		Adjacent organ invasion	Absence	119	112	39	270	0.361
	IIA	11	20	4	35			Presence	10	17	5	32	
	IIB	32	38	9	79		LNR	0	83	61	29	173	0.036
	III	17	36	7	60			0–0.16	26	32	8	66	
Recurrence patterns	Absence	129	24	21	174	<0.001		>0.16	20	36	7	63	
	Local	0	29	10	39		Satellite foci	Absence	123	120	44	287	0.180
	Liver-only	0	43	6	49			Presence	6	9	0	15	
	Lung-only	0	10	2	12		Pancreatic membrane invasion	Absence	81	74	28	183	0.608
	Other sites	0	1	4	5			Presence	48	55	16	119	
	Local + distant	0	13	1	14		PI	0	93	78	28	199	0.310
	Multiple	0	9	0	9			1	31	40	13	84	
LN metastasis	Absence	83	61	30	174	0.007		2	5	11	3	19	
	Presence	46	68	14	128		Imaging tumor size (cm)	≤ 2	63	30	11	104	<0.001
LN5 metastasis	Absence	127	129	44	300			2–4	45	68	28	141	
	Presence	2	0	0	2			>4	21	31	5	57	
LN6 metastasis	Absence	126	128	44	298	0.391	Imaging LN metastasis	Absence	73	75	27	175	0.856
	Presence	3	1	0	4			Presence	56	54	17	127	
LN7 metastasis	Absence	128	126	42	296	0.283	Imaging vascular invasion	Absence	106	90	38	234	0.018
	Presence	1	3	2	6			Presence	23	39	6	68	
LN8 metastasis	Absence	126	126	42	294	0.698	Imaging LN size (cm)	≤ 0.5	72	76	29	177	0.715
	Presence	3	3	2	8			0.5–1	30	28	6	64	
LN9 metastasis	Absence	125	125	42	292	0.885		>1	27	25	9	61	
	Presence	4	4	2	10		NLR	≤ 3.32	89	79	29	197	0.423
LN10 metastasis	Absence	127	125	43	295	0.710		>3.32	40	50	15	105	
	Presence	2	4	1	7		dNLR	≤ 3.32	39	42	19	100	0.284
LN11 metastasis	Absence	126	124	44	294	0.367		>3.32	90	87	25	202	
	Presence	3	5	0	8		PLR	≤ 98.13	17	13	6	36	0.692
LN12 metastasis	Absence	116	111	41	268	0.370		>98.13	112	116	38	266	
	Presence	13	18	3	34		PNI	0	31	26	8	65	0.633
LN13 metastasis	Absence	103	92	36	231	0.181		1	98	103	36	237	
	Presence	26	37	8	71		SII	≤ 1000	90	86	30	206	0.867
LN14 metastasis	Absence	122	117	42	281	0.375		>1000	39	43	14	96	
	Presence	7	12	2	21		mGPS	0	93	81	28	202	0.558
LN15 metastasis	Absence	127	123	44	294	0.367		1	23	33	11	67	
	Presence	2	6	0	8			2	13	15	5	33	
LN16 metastasis	Absence	127	113	44	284	<0.001	WBC	≤ 10	124	115	41	280	0.097
	Presence	2	16	0	18			>10	5	14	3	22	
LN17 metastasis	Absence	124	125	44	293	0.424	ALB (g/L)	≤ 35	19	21	6	46	0.895
	Presence	5	4	0	9			>35	110	108	38	256	
LN18 metastasis	Absence	126	126	44	296	0.593	CRP (ng/L)	≤ 3	93	81	28	202	0.251
	Presence	3	3	0	6			>3	36	48	16	100	
Positive LN number	0	83	61	29	173	0.016	CA19-9 (U/ml)	≤ 35	34	16	9	59	0.018
	1–3	36	46	13	95			>35	95	113	35	243	
	>4	10	22	2	34		CEA (ng/ml)	≤ 5	97	77	31	205	0.026
Tumor size (cm)	≤ 2	48	24	16	88	0.007		>5	32	52	13	97	
	2–4	60	68	18	146		HBV infection	Absence	120	122	41	283	0.866
	>4	21	37	10	68			Presence	9	7	3	19	
Tumor differentiation	Well	0	2	0	2	0.009	Chemotherapy	No	78	58	24	160	0.043
	Moderate	72	55	26	153			Yes	51	71	20	142	
	Poor	57	72	18	147								

*M, month; LN, lymph node metastasis; LNR, lymph node ratio; TNM, tumor–node–metastasis stage; PI, prognostic index; NLR, neutrophil-to-lymphocyte ratio; PLR, platelet-to-lymphocyte ratio; PNI, prognostic nutritional index; SII, systemic immune-inflammation index; mGPS, modified Glasgow Prognostic Score; WBC, white blood cell count; ALB, albumin; CRP, C-reactive protein; CA19-9, carbohydrate antigen 19-9; CEA, carcinoembryonic antigen; HBV, hepatitis B virus*.

## Comparisons of Characteristics Between Early and Late Progression Groups

Apart from 129 patients who were free of tumor progression, 129 and 44 patients were included in the early and late tumor progression groups, respectively. As shown in Table 1, the distribution of clinical factors including age, gender, and inflammation-based indexes, was balanced between these three groups, while higher CA19-9 and CEA levels were positively associated with early tumor progression. In terms of pathological factors, patients in the early progression group were more likely to have LN metastases as well as large and poorly differentiated tumors. Significantly large proportions of patients in the early progression group had LN16 metastases, imaging-detected vascular invasion, and more advanced stages of TNM. Additionally, compared with patients in the late progression group, those in the early progression group were more likely to have liver metastases and local recurrence.

### Comparisons of PPS Stratified by Different Progression Patterns

**Figure 1:**
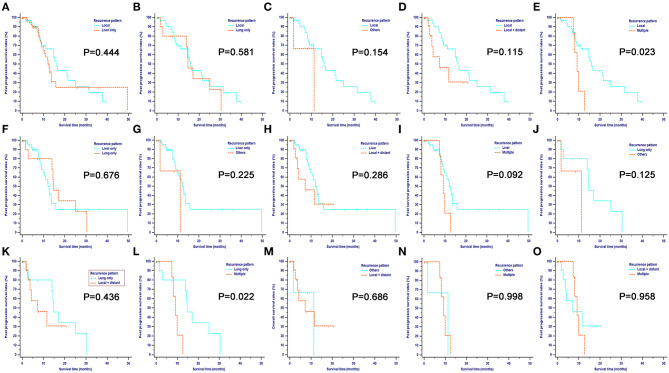
Pairwise comparison of post-progression survival for different tumor progression patterns. Stratification of patients by comparing the following patterns of progression: local vs. liver only **(A)**; local vs. lung only **(B)**; local vs. others **(C)**; local vs. local + distant **(D)**; local vs. multiple **(E)**; liver only vs. lung only **(F)**; liver only vs. others **(G)**; liver only vs. local + distant **(H)**; liver only vs. multiple **(I)**; lung only vs. others **(J)**; lung only vs. local + distant **(K)**; lung only vs. multiple **(L)**; others vs. local + distant **(M)**; others vs. multiple **(N)** and local + distant vs. multiple **(O)**. Landmark analysis was used to analyze survival differences whose survival curves were crossed. For the comparisons of survival rates between local recurrence and other sites, liver-only metastasis and multiple metastases, lung-only metastasis, and multiple metastases, the former had significantly higher survival rates (*P* < 0.05), compared with the latter after 1 year since tumor progression, which was used as the landmark point for survival analyses. Also, patients with local progression had significantly higher survival rates compared with those with multiple analyses while survival rates were similar between other comparison groups. Overall, multiple metastases contributed to the poorest survival among these progression patterns.

Overall, there were six different types of tumor progressions for PDAC patients after surgery. Liver-only metastasis was the most common progression type, followed by local recurrence, local and distant progression, and lung-only metastasis. Metastases at other sites and multiple metastases occupied a small proportion of tumor progressions. The median PPS for all patients was 13.53 months (95% CI 11.24–15.83), and the 1-, 2-, and 3-year PPS rates were 55.9, 26.4, and 10.7%, respectively. Patients with different progression patterns had varied survival rates. As shown in Figure 1, patients with local recurrence had the longest median PPS of 15.93 months (95% CI 11.07–25.03), followed by patients with lung-only metastasis (median PPS 14.7 months, 95% CI 14.00–30.43) and liver-only metastasis (median PPS 12.6 months, 95% CI 9.83–15.77). Landmark analysis was used to analyze survival differences when the survival curves were crossed. The comparisons of survival rates between local recurrence and other sites, between liver-only metastasis and multiple metastases, and between lung-only metastasis and multiple metastases revealed that the former had significantly higher survival rates (*P* < 0.05) than the latter at 1 year following tumor progression (the landmark point for the survival analyses). Further, patients with local progression had significantly higher survival rates than those with multiple analyses, while survival rates were similar between the other comparison groups. Overall, multiple metastases corresponded with the poorest survival rates among these progression patterns.

### Prognostic Factors for PPS

**Figure 2:**
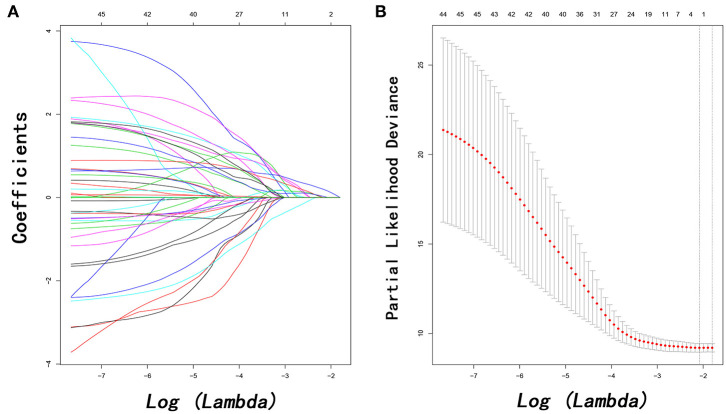
Feature selection using the least absolute shrinkage and selection operator (LASSO) Cox regression model. LASSO coefficient profiles of 48 variables against the log (Lambda) sequence for PPS **(A)** and tuning parameter (Lambda) selection in the LASSO model used 10-fold cross-validation via minimum criteria for PPS **(B)**. PPS, post-progression survival.

In order to investigate prognostic factors for PPS, a LASSO-penalized Cox regression analysis was performed based on 48 high-dimensional radiological and pathological data to further reduce the number of factors in the selected panel with the best predictive performance using the 10-fold cross-validation (Figure 2). Nine variables were selected for PPS prediction by the LASSO-Cox regression model, including LN9 metastasis, LN14 metastasis, LN16 metastasis, local recurrence, liver metastasis, lung metastasis, multiple metastases, tumor differentiation, and imaging-detected tumor size. These predictors, alone with the associated clinical variables identified by univariate analysis, were incorporated in the multivariate analysis. Independent prognostic factors for PPS in PDAC patients following surgery included CA19-9 (HR = 2.524, 95% CI 1.002–6.359, *P* = 0.050), LN9 metastasis (HR = 1.351, 95% CI 1.092–3.430, *P* = 0.042), LN14 metastasis (HR = 1.304, 95% CI 1.074–1.944, *P* = 0.042), LN16 metastasis (HR = 2.785, 95% CI 1.736–10.534, *P* = 0.031), tumor differentiation (HR = 0.492, 95% CI 0.248–0.974, *P* = 0.042), imaging-detected tumor size (HR = 1.579, 95% CI 1.187–2.371, *P* = 0.043), local progression (HR = 5.952, 95% CI 1.869–18.868, *P* = 0.003), liver-only metastasis (HR = 6.452, 95% CI 1.919–21.739, *P* = 0.003), lung-only metastasis (HR = 4.405, 95% CI 1.869–18.868, *P* = 0.046), and multiple metastases (HR = 3.578, 95% CI 1.147–15.887, *P* = 0.042) (Table 2).

**Table 2:** Independent prognostic factors for PPS.

**Characteristics**	**Levels**		**PPS**					
			**Univariate analysis**			**Multivariate analysis**		
			**HR**	**95% CI**	***P***	**HR**	**95% CI**	***P***
Age	≤ 60 years	123	Reference		0.819			NI
	>60 years	104	0.942	0.563–1.576				
Gender	Male	90	Reference		0.088			NI
	Female	137	0.640	0.384–1.068				
WBC	≤ 10	210	Reference		0.010	Reference		0.054
	>10	17	2.488	1.242–4.983		6.125	0.967–38.805	
NLR	≤ 3.32	62	Reference		0.527			NI
	>3.32	165	1.193	0.690–2.063				
dNLR	≤ 3.32	76	Reference		0.215			NI
	>3.32	151	1.399	0.823–2.380				
PLR	≤ 98.13	27	Reference		0.307			NI
	>98.13	200	0.676	0.319–1.434				
PNI	0	49	Reference		0.481			NI
	1	178	1.277	0.647–2.522				
SII	≤ 1000	155	Reference		0.173			NI
	>1000	72	1.505	0.836–2.709				
mGPS	0	152	Reference					NI
	1	50	1.072	0.501–2.296	0.857			
	2	25	1.198	0.494–2.909	0.689			
PI	0	149	Reference			Reference		
	1	64	0.435	0.201–0.944	0.035	3.090	0.424–22.525	0.266
	2	14	0.384	0.161–0.920	0.032	2.863	0.447–18.341	0.267
ALB (g/L)	≤ 35	35	Reference		0.815			NI
	>35	192	1.085	0.549–2.143				
CRP (ng/L)	≤ 3	152	Reference		0.887			NI
	>3	75	1.039	0.612–1.762				
CA19-9 (U/ml)	≤ 35	44	Reference		0.009	Reference		0.050
	>35	183	2.719	1.279–5.780		2.524	1.002–6.359	
CEA (ng/ml)	≤ 5	154	Reference		0.941			NI
	>5	73	0.980	0.581–1.654				
HBV infection	Absence	213	Reference		0.445			NI
	Presence	14	1.577	0.490–5.080				
Chemotherapy	No	120	Reference		0.584			NI
	Yes	107	1.165	0.675–2.010				
Time period to recurrence (month)	>24	14	Reference					NI
	≤ 6	54	4.085	0.864–19.308	0.076			
	6–12	43	3.244	0.766–13.748	0.110			
	12–24	20	2.405	0.569–10.171	0.233			
LN9 metastasis	Absence	219				Reference		0.042
	Presence	8				1.351	1.092–3.430	
LN14 metastasis	Absence	211				Reference		0.038
	Presence	16				1.304	1.074–1.944	
LN16 metastasis	Absence	213				Reference		0.031
	Presence	14				2.785	1.736–10.534	
Tumor differentiation	Well	2				Reference		
	Moderate	115				0.569	0.051–6.305	0.646
	Poor	110				0.492	0.248–0.974	0.042
Pathological tumor size (cm)	≤ 2	66				Reference		
	2–4	110				2.058	0.608–6.960	0.246
	>4	51				1.097	0.370–3.251	0.867
Imaging tumor size (cm)	≤ 2	78				Reference		
	2–4	106				1.579	1.187–2.371	0.043
	>4	43				0.840	0.461–1.531	0.569
Local progression	Absence	30				Reference		0.003
	Presence	197				5.952	1.869–18.868	
Liver-only metastasis	Absence	37				Reference		0.003
	Presence	190				6.452	1.919–21.739	
Lung–only metastasis	Absence	9				Reference		0.046
	Presence	218				4.405	1.869–18.868	
Other metastases	Absence	4				Reference		0.583
	Presence	223				0.590	0.090–3.872	
Local + distant metastasis	Absence	11				Reference		0.377
	Presence	216				0.516	0.119–2.240	
Multiple metastases	Absence	7				Reference		0.042
	Presence	220				3.578	1.147–15.887	
Microvascular invasion	Absence	155				Reference		0.533
	Presence	72				1.237	0.634–2.416	
Imaging vascular invasion	Absence	176				Reference		0.255
	Presence	51				0.519	0.195–1.542	
Imaging LN size (cm)	≤ 0.5	133				Reference		
	0.5–1	48				0.566	0.258–1.242	0.156
	>1	46				0.914	0.360–2.325	0.851
LNR	0	130				Reference		
	0–0.16	50				0.502	0.223–1.130	0.096
	>0.16	47				0.447	0.185–1.080	0.074

*PPS, post-progression survival; HR, hazard ratio; CI, confidence interval; NI, not include; other abbreviations as in Table 1*.

### Construction and Validation of Nomogram for PPS Prediction

**Figure 3:**
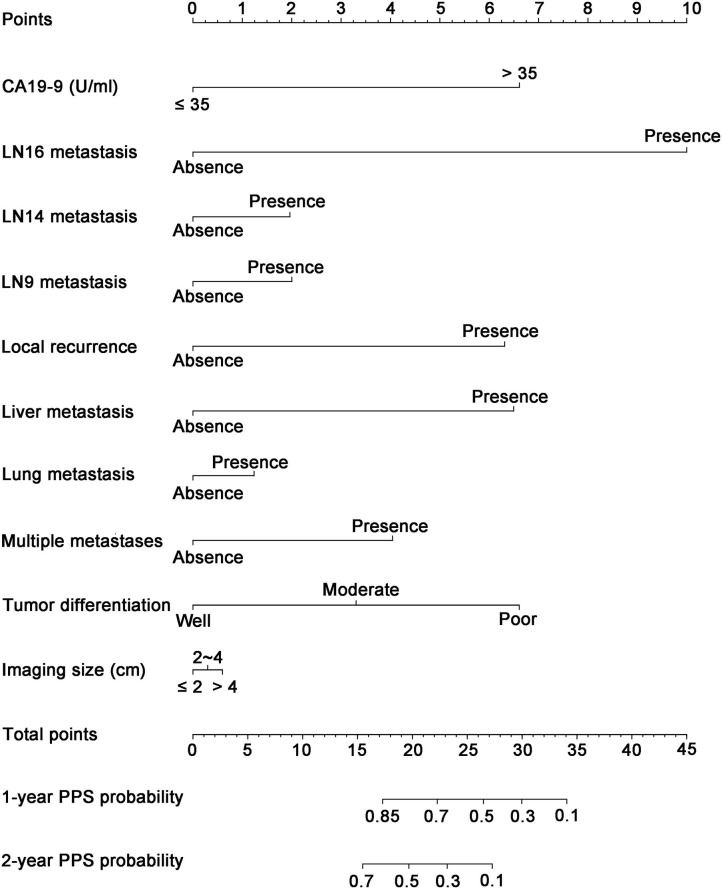
Nomogram for predicting the 1- and 2-year post-progression survival rates in patients with post-operative recurrence of PDAC. PDAC, pancreatic ductal adenocarcinoma.

**Figure 4:**
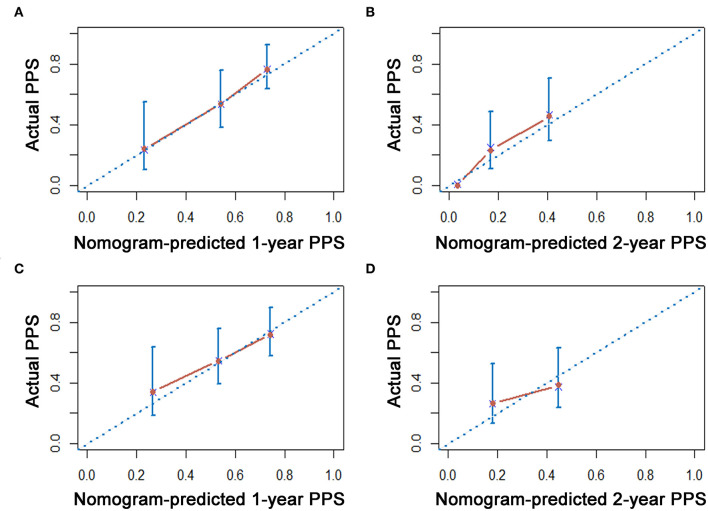
The calibration curve for predicting patient survival at 1 year and 2 years in the training cohort **(A,B)** and validation cohorts **(C,D)**, respectively.

**Figure 5:**
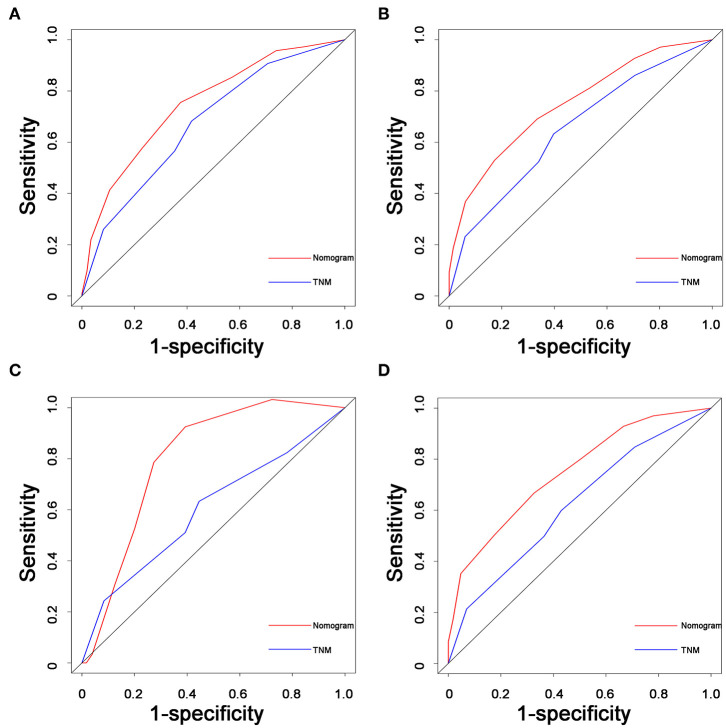
Comparisons of receiver operating characteristic (ROC) curves of both the nomogram and TNM stage system for predicting 1- and 2-year PPS in the training cohort **(A,B)** and validation cohorts **(C,D)**, respectively. TNM, tumor–node–metastasis; PPS, post-progression survival; PDAC, pancreatic ductal adenocarcinoma.

As shown in Figure 3, a specific nomogram was built based on independent prognostic factors for PPS. LN16 metastasis demonstrated the most prominent effect in PPS prediction, followed by local recurrence and liver-only metastasis. Calibration plots showed high agreement between predicted and actual survival in both training and validation cohorts (Figure 4). The C-indexes of the nomogram based on the training and validation cohorts were 0.751 (95% CI 0.692–0.0.810) and 0.710 (95% CI 0.645–0.755), respectively; these values were significantly higher than those of the 8th TNM stage system (Table 3). Comparisons of discriminatory capacity between the nomogram and the 8th TNM stage system were conducted using ROC curves (Figure 5). For the training and validation cohorts, the AUC values for 1-year and 2-year PSS rates were 0.745, 0.747, and 0.783, 0.748, respectively; these values were also higher than those of the 8th TNM stage system (Figure 5).

**Table 3:** Comparison of the C-index and AUC values between nomograms and TNM stage.

**System**		**PPS**			
		**C-index**	**AUC**		***P***
			**1-year**	**2-year**	
Training cohort	Nomogram	0.751 (0.692–0.810)	0.745	0.747	<0.001
	TNM stage	0.602 (0.534–0.680)	0.622	0.618	
Validation cohort	Nomogram	0.710 (0.645–0.775)	0.783	0.748	<0.001
	TNM stage	0.608 (0.536–0.680)	0603	0.619	

*PPS, post-progression survival; TNM, tumor–node–metastasis; AUC, area under receiver operating characteristic curve; C-index, concordance index; other abbreviations as in Table 1*.

## Discussion

Recurrence is an important feature of PDAC after surgery, as it contributes to poor prognosis (7). Previous studies have shown that more than 60% of PDAC patients develop tumor recurrence (12). Similar results were also obtained in the present study, in that the patients in this study experienced a recurrence rate of 57.3%. Given that the survival time of PDAC patients decreases significantly after tumor progression, it is necessary to establish an efficient prognostic system to predict PPS in these patients. Using a large cohort, we developed and validated a novel nomogram based on the characteristics of recurrence, which could be used to accurately stratify patients into distinct prognostic subgroups with significantly different PPS rates.

To date, many studies have consolidated that PDAC is a systemic disease (4, 11). Similar results were also obtained in the present study. In this study, most progressions occurred at the first year following surgery, indicating the systemic nature of this disease. Therefore, exploring the timing and patterns of recurrences is important in survival analyses of PDAC. Additionally, compared with OS, PPS was more influenced by recurrence-related factors as opposed to the characteristics of the primary surgery (13). In this study, most included prognostic factors were related to recurrence. Three additional variables—CA19-9, tumor size, and tumor differentiation—were found to be related to primary tumor status, suggesting that these factors have value for PPS estimation in addition to the effects on tumor recurrence.

Similar to previous studies (4, 7), the present study recorded six different types of recurrence patterns. Liver-only metastasis and local recurrence contributed to the majority of tumor progressions, with most occurring in the early phase. Multiple metastases and distant metastases at sites apart from liver and lungs contributed to only a small proportion of tumor progressions. However, the presence of multiple metastases indicated the poorest PPS for PDAC patients compared with other types of tumor progressions. Patients with local recurrence had the longest median PPS, followed by patients with lung-only and liver-only metastases. Compared with other types of tumor progressions, largeness of the tumor bed capacity and the functional preservation of the lungs or liver in lung or liver metastases were helpful for obtaining longer survival times after tumor progressions. Moreover, lung-only and liver-only metastases shared similar survival rates. A 48 high-dimensional radiological and pathological data was incorporated into the LASSO regression, showing that LN9 metastasis, LN14 metastasis, LN16 metastasis, tumor differentiation, imaging-detected tumor size, local progression, liver-only metastasis, lung-only metastasis, and multiple metastases were independent prognostic factors for PPS in PDAC patients following surgery. Moreover, multivariate analysis showed that CA19-9 was also an independent prognostic factor for PPS in these patients. In the Japanese Pancreas Society staging systems for pancreatic cancer, the para-aortic LN16 is categorized as a Group 3 LN station. LN16 metastasis is considered indicative of distant metastasis and poor survival in PDAC (14). LN16 positivity is common in PDAC, and a standard lymphadenectomy of positive LN16 is helpful in elevating survival and has demonstrated the great impact of LN16 metastasis on PPS in PDAC patients (15). Compared with the other variables included in the present study, LN16 metastasis had the greatest impact on PPS, followed by liver-only metastasis, local recurrence, and multiple metastases. The distant genetic signatures of metastatic lesions might contribute to organ-specific metastases, and the exploration of their mechanisms could potentially illuminate personal therapeutic approaches.

Apart from the progression patterns, increased CA19-9 levels and tumor largeness were important characteristics of high tumor burden in PDAC, which indicated poor treatment response and early progression (16, 17). Poorly differentiated tumors indicated poor survival as well. A previous study indicated that poorly differentiated tumors release certain molecules, including epidermal growth factor and E-cadherin, which could enhance the development of distant metastases and shorten survival times (18). Compared with pathological tumor size, imaging-detected tumor size was more heavily weighted in the survival analysis and was considered an independent prognostic factor for PPS. The calculation or evaluation of the largest tumor size through image comparisons of different levels of tumors with a 1-mm interval was considered comprehensive and accurate. However, the measurement of the largest pathological tumor size was slightly more subjective, as it was nearly impossible to compare tumor sizes from each level of tumors. This may explain the greater role of imaging-detected tumor size compared with pathological size in predicting survival. In addition, the patients included in this study were from 2008 to 2018 and received no neoadjuvant chemotherapy. Following surgery, 142 patients received adjuvant chemotherapy and 160 patients did not receive adjuvant chemotherapy. Moreover, most of the patients were in the relatively early stages of PDAC (TNM I and II); this may explain the insignificance of chemotherapy in the survival analysis. Further evaluation of the prognostic value of chemotherapy in PDAC is needed.

A nomogram for PPS estimation was established based on these independent prognostic factors, which were selected by evaluating high-dimensional radiological and clinicopathological variables. Compared with traditional nomograms for survival prediction among PDAC patients, our nomogram relied on factors related to recurrence and more precisely indicated survival after tumor progression. Additionally, compared with the 8th TNM stage system, the presently developed predictive nomogram showed higher AUC and C-indexes values and stronger predictive power for PPS in both training and validation cohorts. The inclusion of specific indicators of progression patterns in addition to primary tumor characteristics ensured that the nomogram would display better discrimination power. Further, the relatively large cohort size of the present study could have made these results more generalizable than those from single-center studies with smaller numbers of patients. Physicians can use this nomogram to assess a variety of parameters with objectivity and precision and to distinguish between different subgroups of PPS among patients with PDAC following radical resection. Therefore, the presently established nomogram can be used as a practical tool to predict survival after tumor progression and has the potential for use in decision-making regarding the subsequent treatment of PDAC patients following surgery. Apart from the precise prediction of survival rates after tumor progression, the established nomogram had indicated several risk factors after surgery, including LN16, LN9, and LN14 metastases, poor tumor differentiation, and higher levels of CA19-9. Patients with these risk factors need to have adjuvant chemotherapy or radiochemotherapy as soon as possible after surgery to prolong survival. Additionally, when recurrence happens, this nomogram indicates that local recurrence and liver metastasis are more likely to lead to poorer survival, compared with lung metastasis. The additional special treatment for recurrence lesions or liver metastasis apart from the conventional chemotherapy, such as tumor ablation, may contribute to better survival for these patients.

The present study had several limitations. First, some variables were unavailable for this study, including specific treatment following surgery as well as the time period and regimen of chemotherapy. The inclusion of these variables could further support the feasibility of the nomogram for use with PDAC patients. Further, it was a limitation for the inclusion of local regression or metastases in that it neglected their time-related nature. Second, it is expected that more tumor progressions would be observed if the follow-up period were extended. Although all the patients were followed for more than 1 year, a longer follow-up period is needed for a more precise overview of tumor progression following surgery. Third, although neoadjuvant chemotherapy is an important factor that may have impacted prognosis, it was not included in the present analysis. Although good fitness was demonstrated for validation in the present study, we should recognize that bootstrapping is only helpful in reducing the overfit bias of the nomogram. More validations using large, independent cohorts are necessary for the validation of the present nomogram.

In conclusion, we compared the PPS of different progression patterns and established a nomogram to predict PPS in patients with postoperative recurrence of PDAC. Validation based on training and validation cohorts showed that this nomogram has great predictive power for survival. The exploration of risk factors and the establishment of this nomogram could illustrate new versions of personalized recurrence management for PDAC patients following surgery.

## Data Availability Statement

The raw data supporting the conclusions of this article will be made available by the authors, without undue reservation.

## Ethics Statement

The studies involving human participants were reviewed and approved by the Institutional Review Board of Sun Yat-sen University Cancer Center. The patients/participants provided their written informed consent to participate in this study. Written informed consent was obtained from the individual(s) for the publication of any potentially identifiable images or data included in this article.

## Author Contributions

SL was responsible for the conception, design, and quality control of this study, reviewed and edited the manuscript. CH, SS, and YZ performed the study selection, data extraction, and statistical analyses and were major contributors in writing the manuscript and contributed to the writing of the manuscript. CH and SS participated in study selection and statistical analyses. CH, SS, YZ, and XL contributed in classification criteria discussion. All authors have read and approved the final version of the manuscript.

## Conflict of Interest

The authors declare that the research was conducted in the absence of any commercial or financial relationships that could be construed as a potential conflict of interest.
